# Hypertensive Crisis and Myocardial Infarction With Non-obstructive Coronary Arteries in a Leprosy Patient With Erythema Nodosum Leprosum: The Role of Corticosteroids

**DOI:** 10.7759/cureus.77041

**Published:** 2025-01-06

**Authors:** Nishant Rathod, Saket S Toshniwal, Roma Chavhan, Sourya Acharya, Anjalee Chiwhane

**Affiliations:** 1 General Medicine, Datta Meghe Institute of Higher Education and Research, Wardha, IND

**Keywords:** coronary angiography, corticosteroids, hypertension, leprosy, minoca

## Abstract

A hypertensive crisis is a severe condition characterized by a sudden, critical rise in blood pressure, which can lead to organ damage. Myocardial infarction occurring in the absence of significant coronary artery stenosis is referred to as myocardial infarction with non-obstructive coronary arteries (MINOCA). Patients with leprosy may develop erythema nodosum leprosum (ENL), a serious inflammatory condition that can impair cardiovascular health and is frequently treated with corticosteroids. In this case, we describe a 52-year-old male patient, known to have hypertension, who complained of breathlessness, facial edema, and chest pain. Additional testing indicated hypertension, elevated cardiac biomarkers, and an ECG that showed characteristics of a non-ST segment elevation myocardial infarction (NSTEMI). When coronary angiography was performed, non-obstructive epicardial coronary arteries were discovered.

## Introduction

The management of inflammatory and autoimmune disorders, such as erythema nodosum leprosum (ENL), a complication of leprosy characterized by painful nodules and systemic inflammation, is primarily dependent on steroid medication. Although steroids are a useful tool for treating ENL and other disorders, their use carries significant risks, particularly concerning cardiovascular health. One such danger is their ability to trigger a hypertensive crisis, especially in individuals who already have hypertension [1].

Hypertension, or high blood pressure, is a common comorbidity in many patients requiring steroid treatment. The hypertensive effects of steroids are well-documented and can exacerbate pre-existing hypertension or even precipitate severe hypertension in individuals with previously normal blood pressure levels. This can lead to acute complications, including a hypertensive crisis, characterized by severely elevated blood pressure levels that can cause end-organ damage [2,3]. A hypertensive crisis in a patient already suffering from hypertension can be particularly perilous. Acute cardiovascular events, such as myocardial infarction, are more likely to occur under such conditions. Myocardial infarction with non-obstructive coronary arteries (MINOCA) is one symptom of these occurrences. MINOCA, frequently caused by various underlying pathophysiological conditions, occurs if symptoms of myocardial infarction manifest without coronary artery blockage [4].

Due to the extensive use of angiography after acute coronary syndrome, the prevalence of MINOCA has increased. However, its prevalence varies widely, ranging from 3.5% to 15% [5]. Many deaths worldwide are due to atherosclerosis and coronary artery disease, with myocardial infarction and congestive heart failure being the main causes. Atherosclerosis develops due to the accumulation of lipids over time and repeated damage to the blood vessel's inner lining [6].

This case revolves around the complex connection between the management of ENL, steroid-induced hypertensive urgency, and its progression to MINOCA. By understanding this, healthcare professionals can better manage patients requiring steroid therapy and mitigate the effects in hypertensive patients. Through this approach, complications can be avoided.

## Case presentation

A 52-year-old male presented to the hospital with complaints of chest pain, which was subacute in onset, progressive, and diffuse in nature, radiating to the back and left arm for the past 15 days. He had a one-month history of breathlessness and swelling all over his face. The facial swelling was insidious in onset, gradually progressive, and not associated with pain or itching. The breathlessness, which was insidious in onset and gradually progressive in nature, aggravated on exertion and was graded as class II in the New York Heart Association (NYHA) classification. Additionally, he had abdominal striae all over his abdomen, which mimicked Cushing's syndrome.

Four months earlier, the patient presented to the dermatology outpatient department with complaints of hypopigmented skin patches and loss of sensation for the past two months. A skin smear for acid-fast bacilli was sent, which returned positive, confirming the presence of Mycobacterium leprae. A skin biopsy was also taken for examination, showing granulomatous inflammation with numerous acid-fast bacilli. Through this, a diagnosis of multibacillary leprosy was made, and the patient was started on multidrug therapy (MDT) with Rifampicin at 600 mg once monthly, clofazimine at 300 mg once monthly and 50 mg daily, and Dapsone at 100 mg daily. After three months of starting MDT, the patient developed multiple painful red nodules on the skin with fever and joint pain. On examination, a blood pressure of 150/90 was noted. Investigation revealed an elevated white blood cell count, and erythrocyte sedimentation rate (ESR) and C-reactive protein (CRP) were elevated. Skin smears of the lesions were sent which came out positive for acid-fast bacilli, supporting the diagnosis of erythema nodosum leprosum (ENL). Other markers of systemic inflammation were also elevated. He was started on prednisolone at a dose of 60 mg once daily. He had a history of hypertension for four years and was on irregular medications for the same. He did not have any history of diabetes mellitus, tuberculosis, bronchial asthma, or thyroid disorders. The patient’s family history included his mother, who also had a history of leprosy ten years ago and took complete treatment for the same. She had no prior history of premature coronary artery disease.

**Figure 1:**
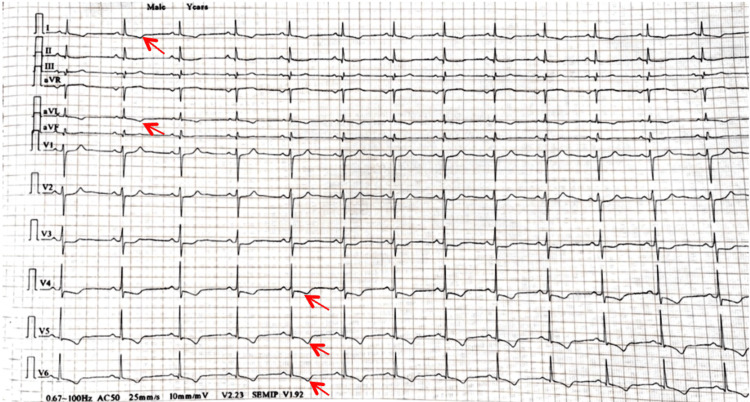
12-lead ECG of the patient showing T wave changes, as indicated by the red arrows. ECG: Electrocardiogram.

On admission, the patient's general condition was apparently stable, and he was well-oriented to time, place, and person. He had a pulse rate of 100 beats per minute, blood pressure of 220/110 mmHg, respiratory rate of 20 breaths per minute, and jugular venous pressure (JVP) was raised. Abdominal examination revealed abdominal striae on inspection, which mimicked Cushing's syndrome; respiratory system examination revealed bilateral crepitations on auscultation, while neurological examination showed no significant abnormalities. Auscultation indicated normal S1 and S2 sounds, and the cardiovascular system demonstrated a normal point of maximum impulse (PMI). There were no murmurs, S3 or rub, but S4 was audible at the apex. An ECG was done, and it revealed T wave inversion in I and aVL leads along with v4-v6 leads, as shown in Figure 1. This, along with raised cardiac biomarkers (ckmb-49, hscTnI-68), was suggestive of ischemic etiology, and hence a diagnosis of NSTEMI with hypertensive crisis was made.

All routine investigations along with cardiac biomarkers were sent, as mentioned in Table 1.

**Table 1:** Laboratory parameters. INR: International normalized ratio; hs-cTnI: High sensitivity cardiac troponin I; CRP: C-reactive protein; ESR: Erythrocyte sedimentation rate; IL-6: Interleukin-6; TNF-alpha: Tumour necrosis factor-alpha.

Lab Parameters	Observed value	Normal range
Haemoglobin	9.9	13-18 gm %
Total leucocyte counts	9000	4000-11000 / cumm
Platelets	2.7	1.5-4 lakh/ cumm
Mean corpuscular volume	84.8	83-101 fl
Urea	18	19 - 43 mg/dl
Creatinine	1.3	0.66-1.25 mg/dl
Potassium	3.8	3.5 – 5.1 mmol/l
Sodium	141	134-145 mmol/l
Alkaline phosphatase	111	38-125 IU/L
Alanine aminotransferase	17	Male < 50 U/L, Female <35 U /L
Total Protein	6.4	6.3-8.3 gm/dl
Total bilirubin	1.3	0.2-1.4 mg / dl
Random blood sugar	128	140-200 mg/dl
Creatinine Kinase Myoglobin Binding	49	9-16
hs-cTnI	68	Male: up to 13 pg/dl Female: up to 9 pg/dl
Serum Calcium	8.2	8.4-10.2 mg/dl
Serum Magnesium	1.9	1.6-2.3 mg/dl
INR	1.22	1.0-1.2
Cortisol	2.86	4.46-22.7 mcg/dl (8am)
CRP	30	<1mg/dl
ESR	62	<20mm/hr
IL-6	53	1-7 pg/ml
TNF-alpha	75	1-8pg/ml

**Figure 2:**
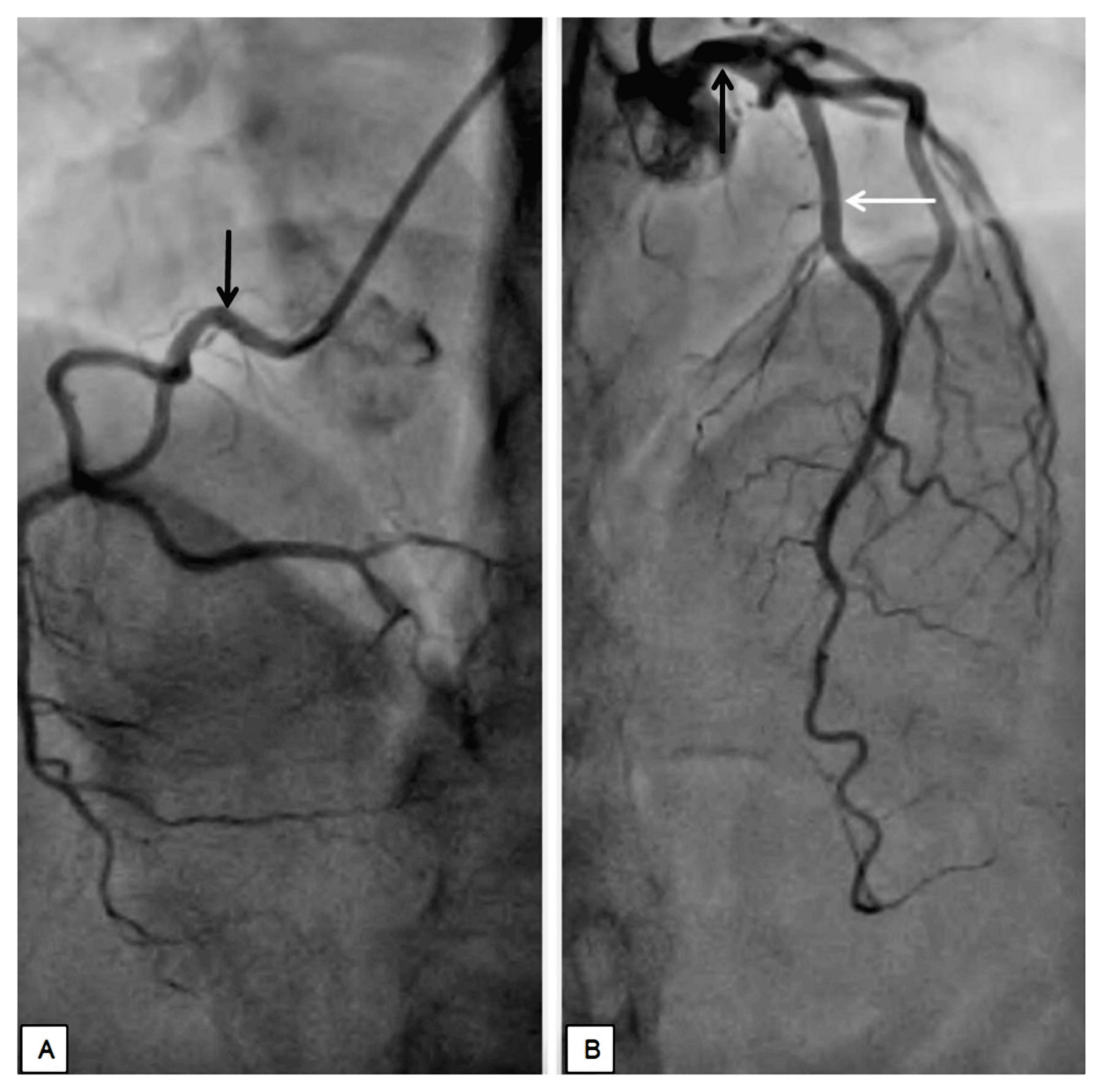
Coronary angiogram: A) depicting normal RCA (black arrow); B) depicting normal LMCA (black arrow) and normal LAD (white arrow). RCA: Right Coronary Artery; LMCA: Left Main Coronary Artery; LAD: Left Anterior Descending Artery.

On echocardiogram, a concomitant regional motion abnormality with mild left ventricular dysfunction with an overall reduction in left ventricular ejection fraction (LVEF) as 45% was noted. A cardiology consultation was taken in view of ECG changes and 2D echo findings, and advice for coronary angiography was given. Coronary angiography was performed, suggestive of normal epicardial coronaries, as shown in Figure 2. Medical management was advised for the same.

Myocarditis and Takotsubo syndrome were considered in the differential diagnosis but were ultimately ruled out. Myocarditis is often associated with preceding viral illness and systemic symptoms like fever and malaise. The absence of these symptoms, or prior febrile illness, reduced the likelihood of myocarditis. The lack of diffuse global dysfunction on echocardiography also argued against this diagnosis. Takotsubo syndrome was also unlikely, as there was no recent history of severe emotional or physical stress, which is a common precipitant. Moreover, echocardiographic findings did not reveal the classic pattern of apical ballooning, thereby ruling out Takotsubo syndrome.

**Figure 3:**
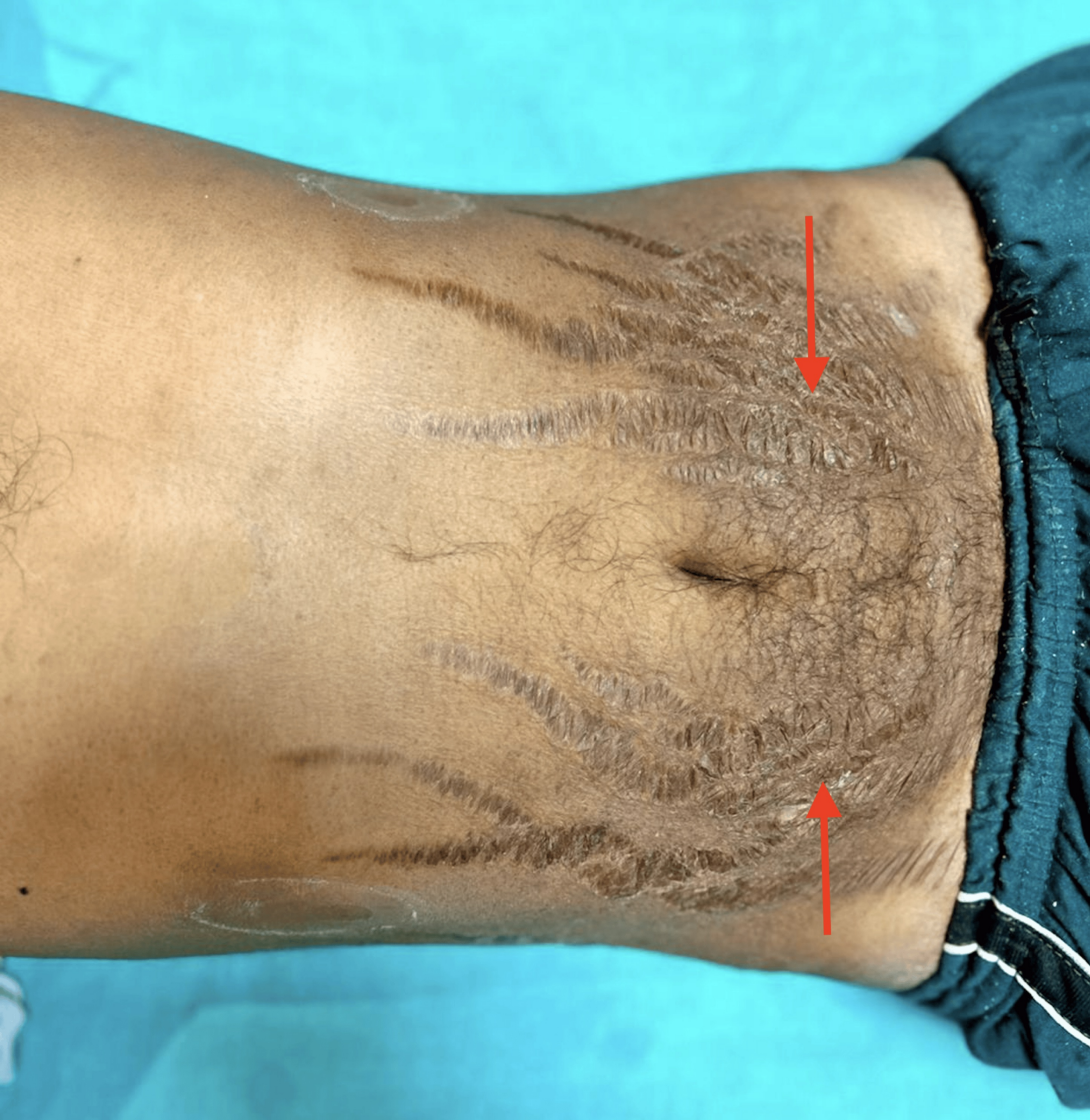
Image depicting abdominal striae (red arrows), which are a typical feature of Cushing's syndrome.

**Figure 4:**
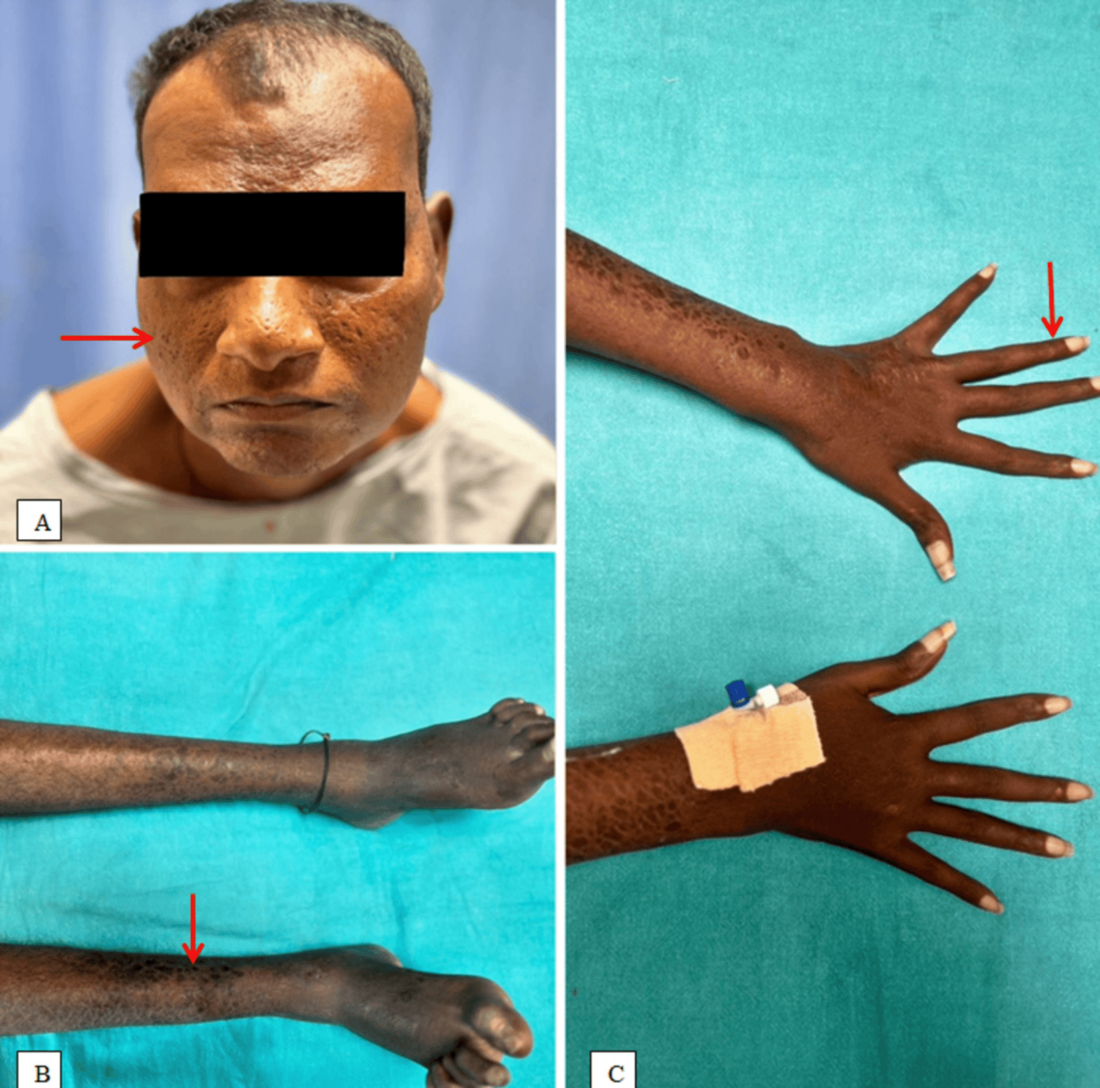
Cushingoid features: A) Moon face (red arrow); B) Skin fragility (red arrow); C) Slender fingers (red arrow).

An Endocrinology consultation was taken in view of cushingoid-like features as shown in Figures 3-4, and a diagnosis of exogenous Cushing's (steroid-induced suppressed cortisol) was made due to long-term use of steroids, and he was advised a low dose dexamethasone suppression test to rule out Cushing's syndrome. A low-dose dexamethasone test was performed, which came out to be negative as the cortisol levels were reduced (1.65) from the baseline levels (2.86), which was suggestive of a normal study.

Upon admission, the Acute Coronary Syndrome protocol was initiated, and the loading dose was given to the patient after NSTEMI was diagnosed, which included Tab ecosprin 300 mg, tab clopidogrel 300 mg, and tab atorvastatin 80 mg, after which the patient was started on dual antiplatelet therapy with tablet ecosprin 75 mg od, tablet clopidogrel 75 mg od, and tab atorvastatin 40 mg hs as well as initiation of low molecular weight heparin 0.6 ml subcutaneous once a day for a period of 5 days. As soon as the patient was admitted to the medical inpatient hospital, the patient was started on inj nitroglycerin infusion at a rate of 0.6 ml/hr and continuous blood pressure monitoring was maintained. On the third day of admission, after the chest pain was resolved, the patient was shifted to the general ward from the intensive care unit as he was stable vitally and his hospital stay was uneventful. A few days later he was discharged on Aspirin, clopidogrel, atorvastatin, amlodipine, atenolol, and sublingual nitroglycerin as needed to follow up in the outpatient clinic.

As the patient was on steroid therapy for more than three weeks, it was gradually discontinued over a period of two months after gradually tapering the dose to avoid Addisonian crisis or acute adrenal insufficiency, and a steroid-sparing agent such as thalidomide was introduced at an initial dose of 100 mg/day.

**Figure 5:**
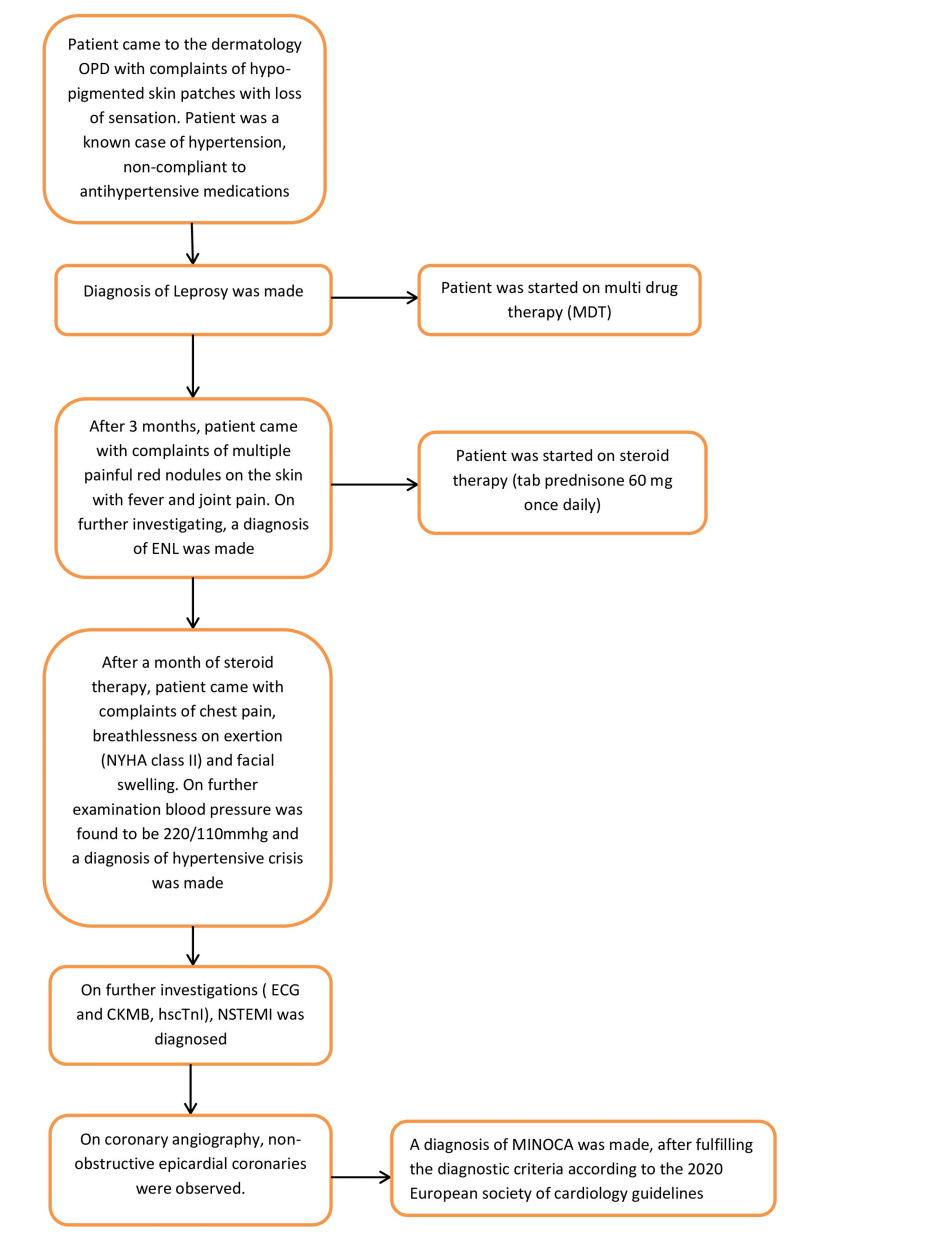
Summary of the timeline of events leading to MINOCA. ENL: Erythema nodosum leprosum; NYHA: New York Heart Association; CKMB: Creatine kinase myoglobin binding; hs-cTnI: High sensitivity cardiac troponin I; NSTEMI: Non-ST segment elevation myocardial infarction; MINOCA: Myocardial infarction with non-obstructive coronary arteries.

A detailed timeline regarding the sequence of events has been mentioned in Figure 5.

## Discussion

High-dose corticosteroids, particularly prednisone, are often necessary for managing systemic inflammation in patients with ENL, a severe complication of leprosy. Although corticosteroids are effective in reducing the initial inflammatory response in ENL, they also pose a significant risk to the cardiovascular system, especially in patients with pre-existing diseases or a predisposition to hypertension. This case demonstrates the dual nature of corticosteroid medication, which can alleviate symptoms while also exacerbating serious cardiovascular issues, such as hypertensive crisis and MINOCA [7].

Prednisone, frequently used to treat ENL, has several dose-dependent side effects, including metabolic disruptions, fluid retention, and hypertension. Blood volume and blood pressure are elevated as a result of sodium and water retention through activation of the renin-angiotensin-aldosterone system (RAAS). Specifically, corticosteroids enhance aldosterone secretion from the adrenal glands, playing a key role in the RAAS pathway. These effects occur largely due to their mineralocorticoid activity. In patients with pre-existing cardiovascular conditions, this activation exacerbates heart failure, increases afterload, raises myocardial oxygen demand, and can also lead to adverse cardiac remodeling. Prednisone also increases the sensitivity of blood vessels to catecholamines, further exacerbating hypertension. Steroids with strong mineralocorticoid effects, like fludrocortisone and hydrocortisone, cause the most fluid retention, while those with less mineralocorticoid activity, such as dexamethasone, triamcinolone, and betamethasone, cause minor fluid retention. Corticosteroid-induced hypertension can be managed with diuretics, and the smallest effective dose and shortest duration of therapy should be used to minimize this risk [7,8,9].

The corticosteroid typically utilized for therapy was prednisolone, in conjunction with a suitable multidrug regimen (MDT). Prednisolone is commonly started as a single morning dosage of 40-60 mg/day. Patients on continuous corticosteroid medication frequently develop Cushingoid features, with "moon face" being the most prevalent feature observed in all patients, even after two weeks of corticosteroid treatment, as in this case [10]. Additionally, prednisone alters lipid metabolism, increasing low-density lipoprotein (LDL) and triglycerides while decreasing high-density lipoprotein (HDL) cholesterol, thereby promoting atherosclerosis. These alterations may lead to endothelial dysfunction and an elevated risk of plaque rupture. Even in the absence of severe coronary artery blockage, fluid retention and high blood pressure can strain the heart and potentially result in myocardial infarction. They can also create a supply-demand mismatch, which might trigger type 2 myocardial infarction, characteristic of MINOCA [7].

A supply-demand mismatch in the context of hypertensive crises, coronary artery vasospasm, microvascular dysfunction, and other factors can all contribute to the heterogeneous disease known as MINOCA. Coronary artery vasospasm involves sudden, temporary narrowing of the coronary artery, reducing blood flow. Restricted blood flow may cause myocardial ischemia, leading to the characteristic symptoms of myocardial infarction. Microvascular dysfunction impairs blood flow regulation in small coronary vessels, resulting in insufficient oxygen supply to the heart muscle. Even though there was no major coronary artery blockage in this patient, the hypertensive crises brought on by corticosteroid medication likely contributed to myocardial ischemia. Given the underlying microvascular dysfunction, the elevated blood pressure would have worsened coronary perfusion and increased myocardial oxygen demand, potentially resulting in ischemia and subsequent myocardial damage [11,12].

According to the 2020 European Society of Cardiology Guidelines for the Management of Non-ST-Elevation MI, individuals diagnosed with MINOCA are those who have acute MI and meet the following criteria: in accordance with the Fourth Universal Definition of Myocardial Infarction, the subsequent prerequisites need to be fulfilled. Three criteria apply to the clinical presentation: 1) acute MI; 2) non-obstructive coronary arteries on angiography (no stenosis ≥ 50%); and 3) no specific alternative diagnosis. This classification excludes myocarditis and Takotsubo syndrome from the final MINOCA diagnosis [13]. Echocardiography and left ventriculography are part of the initial work-up for MINOCA. These tests can reveal information concerning anomalies in wall motion and ejection fraction. The gold standard for determining the state of the coronary vessels and identifying ACS remains coronary angiography. However, coronary reactivity is not assessed by coronary angiography. As such, there is a significant chance that the cause of the myocardial injury in these patients will go unnoticed [14,15,16].

Dual antiplatelet therapy (DAPT) in MINOCA is chosen primarily to address potential thromboembolic causes, such as microvascular thrombus, by preventing platelet aggregation and reducing the risk of future cardiovascular events. While MINOCA presents without significant coronary artery obstruction, its pathogenesis may involve subtle atherosclerosis, thrombosis, or plaque erosion, similar to ACS, for which DAPT is standard treatment. Although specific guidelines for MINOCA are lacking, DAPT is often used empirically to cover thrombotic mechanisms and improve long-term outcomes, including reducing recurrent myocardial infarction [13].

Individuals suffering from MINOCA present a substantial therapeutic challenge. Each MINOCA subtype's underlying physiopathology is taken into account when designing a treatment plan. Statins and renin-angiotensin system blockers have been shown to help lower mortality in MINOCA patients. A randomized experiment called MINOCA BAT is currently being conducted to assess beta-blockers. Beta-blockers reduce myocardial oxygen demand by lowering heart rate and contractility, helping to alleviate ischemia. They also reduce sympathetic activity, lowering the risk of coronary artery vasospasm or microvascular dysfunction contributing to MINOCA. There is insufficient data to support the therapy of MINOCA; therefore, randomized, controlled trials are necessary to establish a systematic treatment plan [12,17,18].

While corticosteroids are indispensable in managing inflammatory complications like ENL, their impact on cardiovascular health cannot be underestimated. In this case, the therapeutic benefits of prednisone in controlling ENL must be weighed against the risk of inducing hypertensive crises and MINOCA. This necessitates a careful approach to corticosteroid dosing, aiming to use the lowest effective dose for the shortest duration possible. Additionally, concurrent use of antihypertensive medications, such as diuretics or beta-blockers, should be considered to mitigate the cardiovascular side effects of steroids [1].

This case emphasizes the importance of cautionary measures when treating patients with corticosteroids, particularly those with underlying cardiovascular disorders. In individuals such as this, a multidisciplinary approach should be employed to avoid serious complications, especially those affecting the cardiovascular system.

## Conclusions

The multitude of etiologies that can cause MINOCA makes diagnosis and treatment challenging. In this case, the patient’s hypertensive crisis, most likely exacerbated by high-dose corticosteroid therapy for ENL, appears to have been the cause of the MINOCA event. This case highlights the need for close monitoring and careful management of medications in patients with complex comorbidities. Corticosteroids, while effective in managing ENL, likely contributed to the development of a hypertensive crisis, underscoring the need for vigilant blood pressure monitoring in patients receiving prolonged steroid therapy, particularly in those who are known hypertensive. The management of such patients requires a multidisciplinary approach involving cardiologists, dermatologists, and internal medicine specialists to balance the need for corticosteroid therapy against the risks of hypertension and cardiovascular events. Furthermore, investigating the pathophysiology of MINOCA in the context of chronic inflammatory conditions and corticosteroid use will help in developing targeted management strategies.

Serious outcomes like MINOCA and hypertensive crisis can be mitigated with the use of corticosteroid-sparing drugs such as thalidomide, which is one of the most commonly used agents in ENL. While generally well-tolerated from a cardiovascular perspective, it still requires close monitoring.
